# Growth origin of large-scale fiberform nanostructures in He–W co-deposition environment

**DOI:** 10.1038/s41598-023-32621-5

**Published:** 2023-04-03

**Authors:** Kenta Hori, Shin Kajita, Rongshi Zhang, Hirohiko Tanaka, Noriyasu Ohno

**Affiliations:** 1 Graduate School of Enginnering, Nagoya University, Nagoya, 464-8603 Japan; 2 Graduate School of Frontier Sciences, The University of Tokyo, Kashiwa, Chiba 277-8561 Japan; 3 Institute of Materials and Systems for Sustainability, Nagoya University, Nagoya, 464-8603 Japan

**Keywords:** Nanoscale materials, Magnetically confined plasmas

## Abstract

When tungsten (W) is deposited with helium (He) plasma (He–W co-deposition) on W surface, enhanced growth of fiberform nanostructure (fuzz) occurs, and sometimes it grows into large-scale fuzzy nanostructures (LFNs) thicker than 0.1 mm. In this study, different numbers of mesh opening (apertures) and W plates with nanotendril bundles (NTBs), which are tens of micrometers high nanofiber bundles, were used to investigate the condition for the origin of the LFN growth. It was found that the larger the mesh opening, the larger the area where LFNs are formed and the faster the formation tends to be. On NTB samples, it was found that NTBs grew significantly when exposed to He plasma with W deposition, especially when the size of the NTB reached $\sim 0.1$ mm. The concentration of the He flux due to the distortion of the shape of the ion sheath is proposed as one of the reasons to explain the experimental results.

## Introduction

Tungsten (W) is a candidate material for divertor in fusion devices, where high heat and particle loads are expected. In addition to hydrogen isotopes, helium (He) is produced by the nuclear fusion reaction between deuterium and tritium and irradiated to metals, causing morphological changes on their surfaces due to the growth of He bubbles^1,2^. Therefore, understanding the interaction between He and W is important and has been studied intensively. Helium plasma irradiation on a W surface forms fiberform nanostructures called *fuzz*^3,4^ when the temperature and incident ion energies are in the range of 1000–2000 K and above 20–30 eV, respectively^5,6^. The necessary condition for fuzz growth could be satisfied around the strike point in the ITER divertor^7^. There are concerns about fuzz formation in fusion reactors, which leads to the significantly reduced thermal resistance^8^ and increased field electron emission^9–11^. This may lead to the initiation of arcing and the release of large amounts of W^5,12–14^. On the other hand, there are several advantages as plasma facing material. Fuzz reduces the sputtering rate by an order of magnitude^15^ and mitigates the crack formation by pulsed loads^16^. In addition, fiberform nanomaterials have various practical applications including optical applications^17,18^. The practical application of fuzz as photoelectrode and gas sensor has been explored so far^6^. Improved performance of gas sensor for hydrogen gas detection has been demonstrated on oxidized W fuzz^19^. Photocatalytic/photoelectrochemical application has been explored on oxidized W fuzz with methylene blue decomposition^20,21^ and with oxygen evolution reaction (OER)^22,23^.

Although it has been shown that the presence of hydrogen isotopes is not influential in the growth of fuzz^24^ in actual fusion environment, a small amount of additional impurities, including radiator gas species and sputtered W, can significantly change the process. A bundle-like fiber structure called a nano-tendril bundle (NTB) on W was grown when W was exposed to He plasmas with small amounts of argon, neon, and nitrogen^25,26^ or with the ion energy modulation by RF biasing^27,28^. In addition, W particles sputtered from the wall can be simultaneously irradiated with He ions. In such a co-deposition environment, it has been found that fuzz growth is significantly enhanced, and the thickness of the fuzzy layer can be on the order of millimeters^29^, which is two to three orders of magnitude larger than that of conventional fuzzy layer^30^. The millimeter-thick fuzzy layer is called a large-scale fiberform nanostructure (LFN), and it was also found to form with molybdenum^31^ and rhenium^32^.

However, the enhanced growth process was not fully understood. The starting point of LFNs growth always formed at the edge of the W substrate and grew in the direction of the flow of plasma^33^. Adatom diffusion and epitaxial growth on fiber surfaces are probably related to the growth of LFNs^32^. However, no LFN growth occurred when the edges of the material were covered^34,35^. This was also the case with magnetron sputtering, where enhanced fuzzy layer growth was observed with auxiliary W deposition, but there was no LFN growth^36^. In particular, the conditions necessary for the origin of the formation of such a large-scale structure are still not fully understood and need to be clarified.

In this study, W-mesh with different mesh openings was used to investigate the growth origin of LFNs. It was shown that the growth origin could be significantly altered by the mesh openings. The key parameters for the origin of LFNs are discussed. In order to demonstrate the effects of protrusions on the formation of LFNs, W plates with NTBs were formed and exposed to the plasma under the co-deposition conditions. The growth rate of NTBs under the co-deposition conditions is shown in detail.

## Methods

### Plasma irradiation

**Figure 1:**
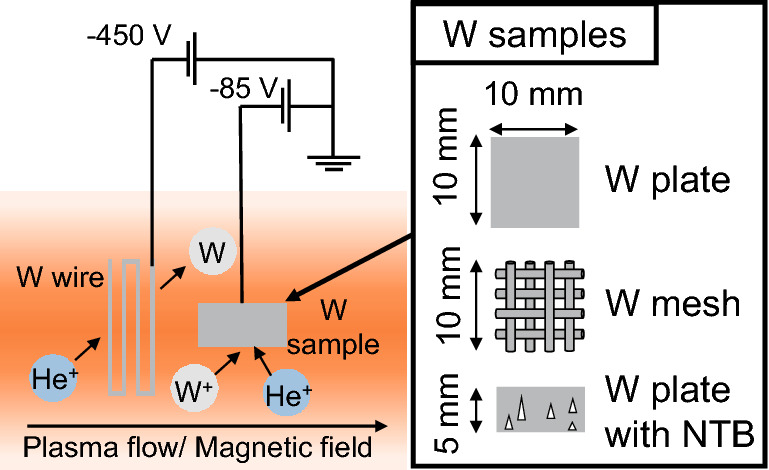
A schematic diagram of the experimental setup.

Figure 1 shows a schematic of plasma irradiation experiments in the He–W co-deposition environment in the linear divertor simulator NAGDIS-II, where the He plasma was generated in a steady state. A W wire ($\phi =0.5$ mm) was placed $\approx 2$ mm adjacent to a W sample, and a bias of − 450 V was applied. Tungsten atoms were sputtered from the W wire by irradiation with high-energy He ions. The sample was irradiated with He ions and W particles (mainly neutrals). In this study, He plasma irradiation with W co-deposition is referred to as He–W co-deposition. The sample was biased to − 85 V. The incident ion energy was 70 eV considering the fact that the space potential was $\approx -$ 15 V. Different types of W were used for the samples: pure W plates ($10\times 10 \times 0.2$ $\hbox {mm}^3$, The Nilaco Co.), W meshes, and NTB-formed W plates ($10\times 10\times 0.2$ $\hbox {mm}^3$). For the W mesh, three types of meshes with different mesh openings (i.e., the distance between the W wires for the mesh) were used (Table 1). The larger the mesh number, the smaller the mesh opening, which is inversely proportional to the mesh number. NTBs were formed by irradiation of He plasma on a W plate ($10\times 5 \times 0.2$ $\hbox {mm}^3$) with a mixture of Ne gas and biasing the W plate to − 250 V. The ratio of Ne pressure was 20%.

**Table 1:** Details of the samples used for co-deposition experiments: mesh number, wire diameter making up the mesh, and mesh opening.

Mesh number	Wire diameter ($\upmu \hbox {m}$)	Mesh opening (mm)
$\sharp 50$	30	0.48
$\sharp 100$	30	0.22
$\sharp 290$	20	0.07

### Observation

**Figure 2:**
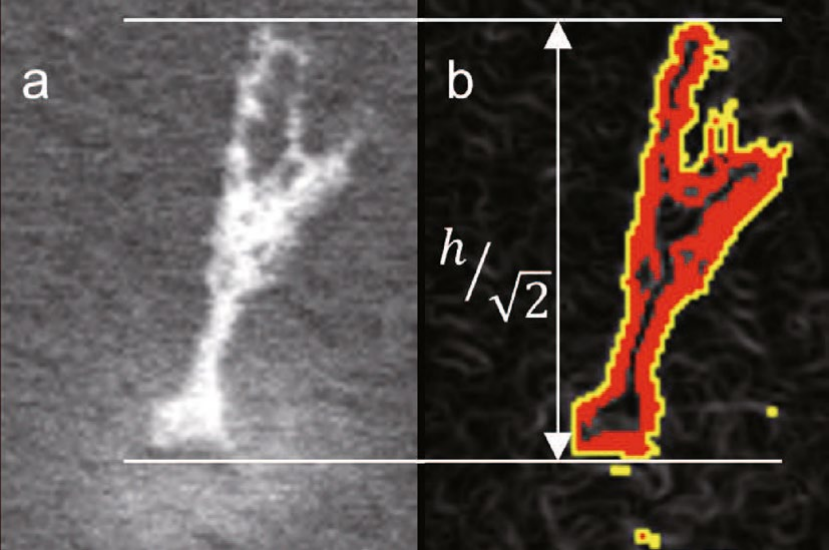
(**a**) A typical SEM micrograph of an NTB, and (**b**) an identified NTB contour after application of the median and Sobel filters and the Otsu method.

The surface changes of the plasma-irradiated samples were observed using an optical camera, a scanning electron microscope (SEM), and a confocal laser scanning microscope (CLSM). In the SEM images, the lengths of the NTBs were measured by analyzing images taken at a $45^\circ$ tilt from the normal, and the height was derived by multiplying by $\sqrt{2}$. Statistical analysis of the optical camera, SEM, and CLSM images was performed using ImageJ software^37^. Figure 2a shows a typical SEM micrograph of an NTB from $45^\circ$. To derive the NTB height from SEM images, the images were filtered by median and Sobel filters^38,39^, and then NTBs were detected by the Otsu method^40^, as shown in Fig. 2b. SEM images may contain sparse impulse noise called salt-and-pepper noise. A median filter is a well-known non-linear filtering technique that can remove impulse-type noise; it is a filter that converts pixel values to the median of neighboring pixels. In this study, a 5 $\times$ 5 kernel was used for filtering. In addition, the Sobel filter was used to enhance the contours. The Otsu method was used to binarize the images to extract NTBs from the SEM images.

CLSM is also used to analyze the height profile of plasma irradiation in NTB-forming samples. The horizontal and vertical laser pitches were set to 2.76 $\upmu \hbox {m}$ and 2.00 $\upmu \hbox {m}$, respectively, and the entire sample surface was observed. Following previous work^26^, structures higher than 6 $\upmu \hbox {m}$ and wider than 114 $\upmu \hbox {m}^2$ were detected as NTBs, respectively, to eliminate noise. It should be noted that the spatial resolution of CLSM is not sufficient to measure the fine tip of NTBs, and it can be used for the average height. SEM images were used to focus on the detailed analysis of the growth of the maximum height of NTBs.

## Results and discussion

### LFN growth on meshes

#### LFN formation

**Figure 3:**
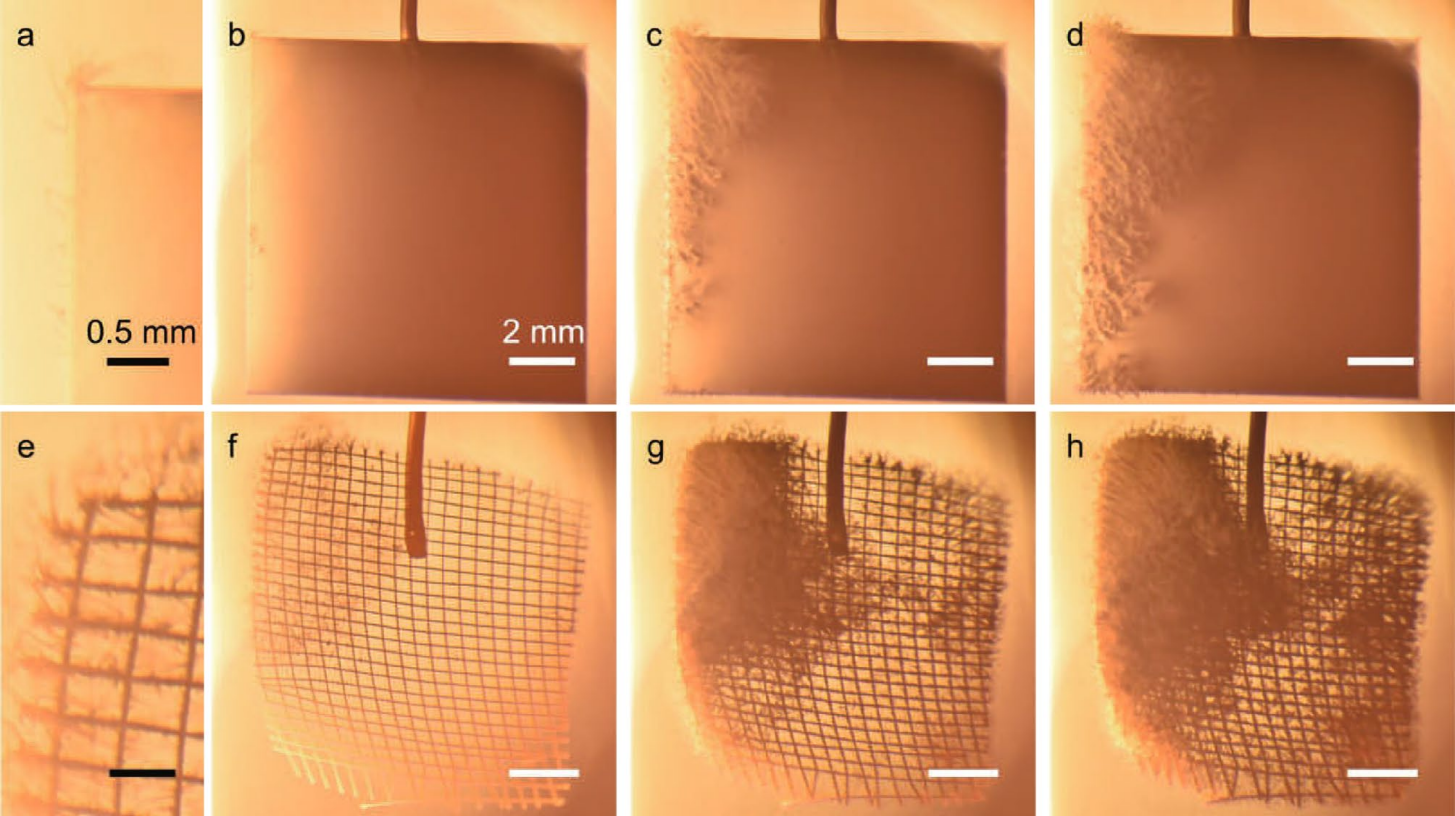
Images representing the formation process of LFNs on (**a**–**d**) the W-plate and (**e**–**h**) on the $\sharp 50$ W mesh, taken by an optical camera during plasma irradiation: (**a**) magnified view of LFN formation on the W plate at 20 min, (**b**–**d**) total view of the W plate at 20, 40, and 60 min, respectively, (**e**) magnified view of LFN formation on the $\sharp 50$ W mesh at 20 min, and (**f**–**h**) total view of the $\sharp 50$ W mesh at 20, 40, and 60 min, respectively.

Figure 3a–d shows images representing the formation process of LFNs on the W-plate, which were captured by an optical camera. LFNs were grown from the left edge of the sample, which is close to the sputtering source, and grew toward the lower right direction, which is consistent with the plasma flow^33^. Figure 3e–h shows pictures representing the formation process of LFNs on the $\sharp 50$ W mesh. After He–W co-deposition, LFNs were formed on the W plate and all kinds of W meshes; the most different results from the W plate were observed on the $\sharp 50$ W mesh. A large number of initiation points were observed even on the downstream (right) side of the mesh.

**Figure 4:**
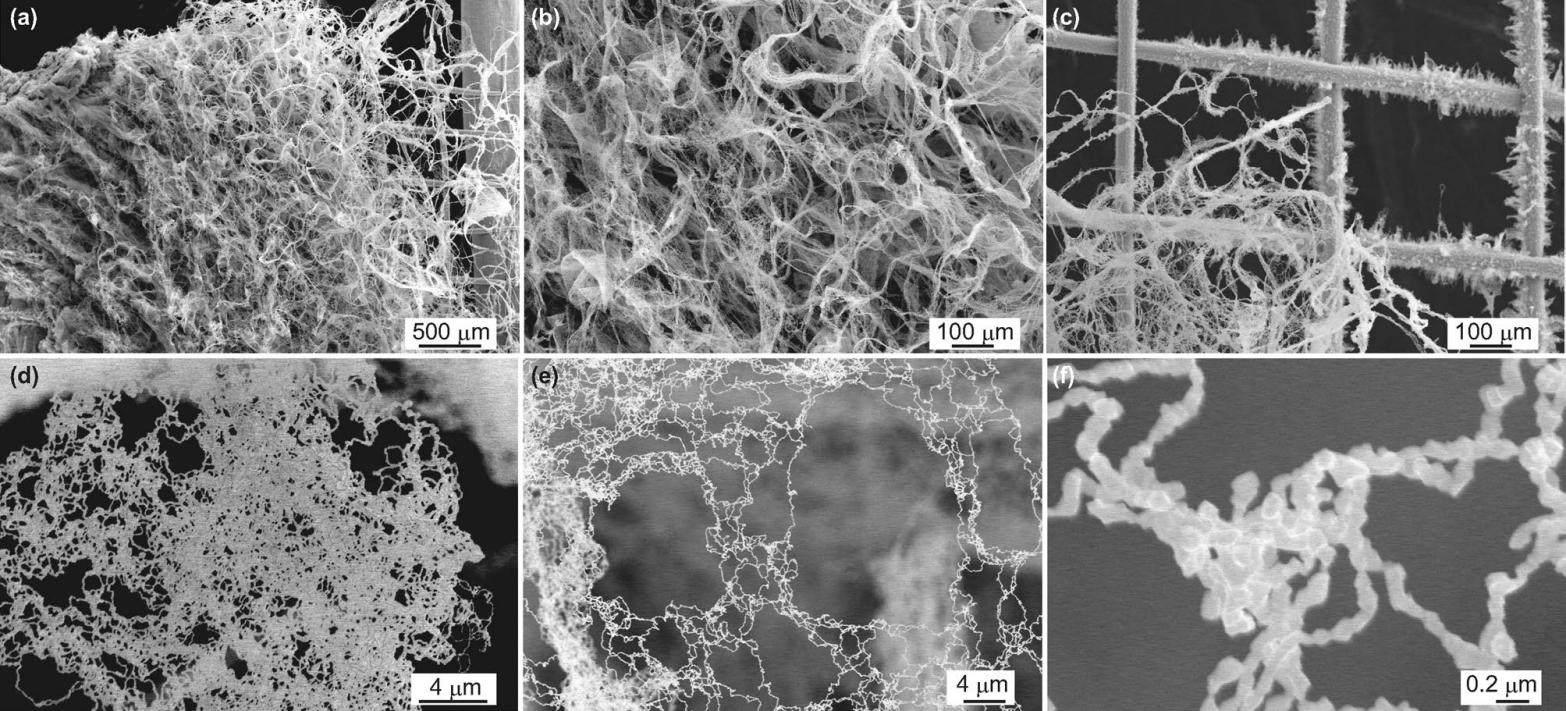
SEM micrographs of LFNs formed on the $\sharp 50$ mesh at various magnifications (at low magnification (**a**–**c**) and high magnification (**d**–**f**)).

Figure 4 shows SEM micrographs of LFNs formed on $\sharp 50$ W mesh at various magnifications. In Fig. 4a, the right edge of the image corresponds to the center top of the mesh, where the mesh was connected to a tantalum wire which was connected to a feed through terminal. The LFNs completely cover the mesh, except at the right edge of the image, where it can be seen that the LFNs are growing out of the mesh. Figure 4b shows the image at a slightly higher magnification at the location where the LFNs are fully grown. It can be seen that the LFNs are composed of woven fine fibers. Figure 4c shows the part where the fibers are grown from the mesh. Fine fuzzy structures with a height of tens of micrometers are grown from the mesh wires on the right, and woven fibers are grown from these fine structures on the lower left. Figure 4d,f shows images of each fiber at even higher magnification. It can be seen that the woven fibers are composed of much finer fibers. In some places the fibers are agglomerated (Fig. 4d), while in other places elongated fibers are connected to form a web-like structure (Fig. 4e). The width of each fiber is 100 nm or less, as shown in Fig. 4f, and the fibers are not straight and have many nodes.

Although the nucleation process of deposited W atoms was not fully understood, it was discussed that adatoms are formed on the surface of nanofibers, and epitaxial growth at the tip of nanofibers plays a key role in the growth of LFNs^32^. There is a clear dependence of the initial morphology change on the crystal orientation^41^; fuzz growth eventually occurs independently of crystal orientation. Each fiber retains a crystal structure, and the crystal orientation changes at the point where the fiber direction changes^42^. It was also found that there is no preferential crystal orientation exists in the axial direction of W fibers grown by He plasma irradiation, although the fibers always grow in the *c*-axis direction of the hexagonal close packed structure for rhenium or ruthenium^32^. It was confirmed by energy dispersive X-ray analysis (EDX) that there are no impurity species in the structure when W fuzz was discovered^3^. Recent XRD study of W fuzz formed on polycrystal W suggested that the grain distribution of the W fuzz layer is similar to that of the bulk layer globally^43^.

**Figure 5:**
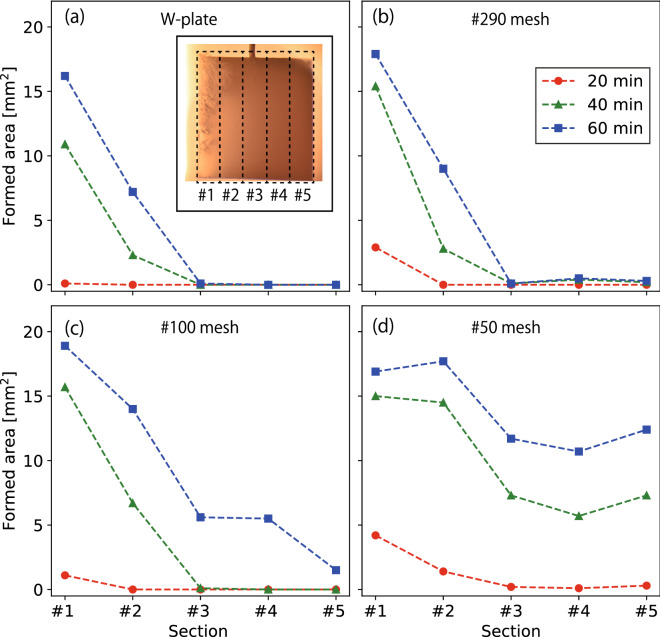
LFN growth area profiles at 20, 40, and 60 min on (**a**) the W-plate, (**b**) $\sharp 290$, (**c**) $\sharp 100$, and (**d**) $\sharp 50$ W-mesh, respectively.

Figure 5a–d shows the profiles of the LFN formed area on the W-plate and $\sharp 290$, $\sharp 100$, and $\sharp 50$ W-mesh at 20, 40, and 60 min, respectively. The sample was divided into 5 sections ($\sharp 1\hbox {-}5$) from the upstream (left) side as shown in the inset in Fig. 5a, and the LFN formed area was measured in each section every 20 min. On the W-plate, the starting point of formation occurred at the upstream edge at 20 min, but the formed area was negligibly small. The LFNs grew as if spreading from the edge as the exposure time was increased to 40 and 60 min. Formation occurred only at $\sharp 1$ and 2, even at 60 min. Growth occurred slightly earlier on the $\sharp 290$ mesh (Fig. 5b), but the difference from the plate was not as significant. On $\sharp 100$ and 50 mesh (Fig. 5c,d), the formed area was not so different at 20 min, but the growth on $\sharp$2–5 was much faster compared to those on the W-plate and $\sharp 290$ mesh. On the $\sharp 50$ mesh, the LFN growth occurred over a wide area including $\sharp 4$ and 5.

**Figure 6:**
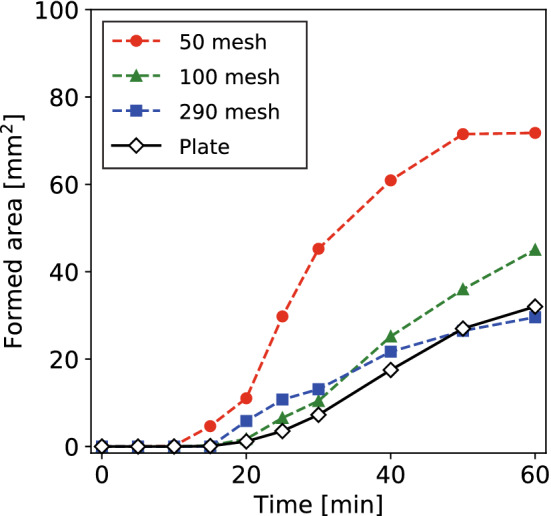
Time evolution of the LFN formed area on the W plate and three types of W-mesh.

Figure 6 shows the time evolution of the total LFN formed area on the W plate and three types of W meshes. On the W plate, the formation started at 20 min, and the LFN formed area was about 30 $\hbox {mm}^2$ at 60 min. The formed area on $\sharp 290$ mesh was almost the same as that of the W plate. About a $\sim 40\%$ increase from the W plate was seen at 60 min on $\sharp 100$ mesh. On the $\sharp 50$ mesh, the formation started at 15 min, and the LFN formed area was about 70 $\hbox {mm}^2$ at 60 min, which was more than twice the area formed on the W plate. Comparing the three meshes, the LFN formed area at 60 min increased with increasing the mesh opening, especially on $\sharp 100$ and 50 meshes.

#### Effect of edges

With He–W co-deposition, the growth of LFNs was investigated using W meshes with different mesh openings and the growth of NTBs was observed in detail. On the one hand, on the $\sharp 290$ W mesh, which has mesh openings of 0.07 mm, the growth characteristics were almost the same as that on the W-plate. On the other hand, on $\sharp 50$ and $\sharp 100$ meshes, which have mesh openings of 0.48 and 0.22 mm, respectively, the growth of LFN occurred from all over the sample. On the W-plate, LFN growth always occurs from the edge of the sample. In other words, we can say that the edge effect occurs over the entire area of $\sharp 50$ and $\sharp 100$ meshes.

**Figure 7:**
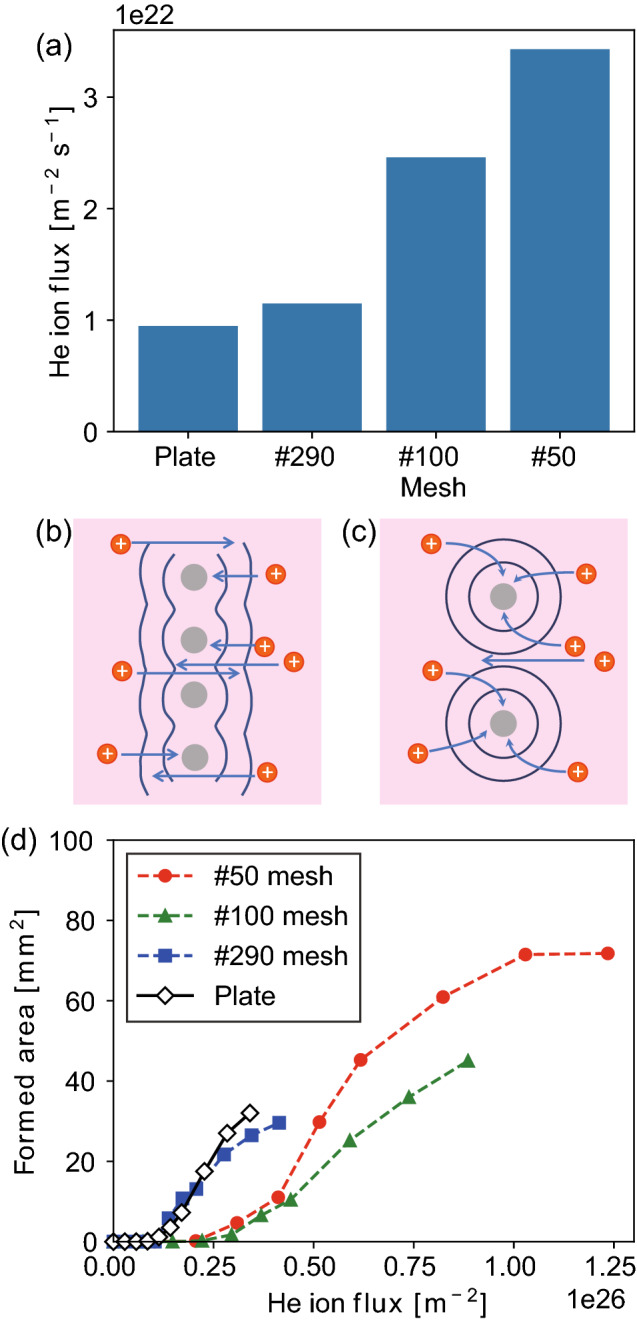
(**a**) Ion flux on the W-plate and W-mesh, schematics of trajectories of ions on the mesh (**b**) with the mesh opening less than the sheath thickness and (**c**) larger than the sheath thickness, and (**d**) LFN formed area as a function of the He ion flux.

At the edges, the ion flux from the plasma will be larger. For example, it has been shown using particle-in-cell (PIC) simulations that the flux at the edge of a castellated target is several tens of percentages greater than the other location and much greater than that within the gap of the castellation^44^. In this work, the ion flux at the edge can be greater than the other locations, because the edge is normal to the surface and can concentrate the flux due to the shape of the ion sheath. A similar situation occurs on meshes. Figure 7a shows the He flux on each sample, which is derived by dividing the sample current by the sample surface area. Here, the geometric surface area of the W wires making up the mesh was used to derive the mesh area. The flux on $\sharp 290$ was $1.2\times 10 ^{22}$ $\hbox {m}^{-2}\,\hbox {s}^{-1}$ and was not that different from that on the W-plate ($9.5\times 10 ^{21}$ $\hbox {m}^{-2}\,\hbox {s}^{-1}$). However, it was much larger on $\sharp 100$ and $\sharp 50$ meshes: 2.6 times on $\sharp 100$ and 3.6 times on $\sharp 50$ compared to that on W-plate.

The flux is likely to be significantly affected by the electric sheath formed around the wires that make up the mesh. From the electrostatic probe measurement, the plasma density and temperature were $2.3\times 10^{18}$ $\hbox {m}^{-3}$ and 4.9 eV, respectively; the Debye length is estimated to be $\lambda _{\textrm{D}}=11~\upmu \hbox {m}$. The Child-Langmuir sheath thickness^45^ can be obtained as

1 $$\begin{aligned} h_{\textrm{CL}}=\frac{\sqrt{2}}{3} \left( \frac{2E_i}{T_e} \right) ^{3/4} \lambda _{\textrm{D}}, \end{aligned}$$

and is calculated to be $\sim 0.06$ mm. For the $\sharp 290$ mesh, half of the mesh openings were less than the sheath thickness. Thus, the sheath formed around the W wires that make up the mesh overlap. In this case, the electric field far from the mesh is mainly in the direction normal to the mesh (Fig. 7b). Thus, since the particles are sufficiently accelerated by the electric field in the direction normal to the grid, the particles would easily pass through the grid without experiencing sufficient attractive force to the wires. For $\sharp 100$ and $\sharp 50$ meshes, since the mesh openings are greater than twice the sheath thickness, the sheath formed around the wire does not overlap. Thus, the ion collection by the electric field formed around the wire works efficiently on $\sharp 100$ and $\sharp 50$ meshes, and the He flux increases on these meshes (Fig. 7c).

The increase in He flux is likely the major contributor to the edge effect that accelerates the growth of LFNs on $\sharp 50$ and $\sharp 100$ mesh. It is noted that the He flux dependence of fuzz growth has been well studied^30^. The thickness of fuzz layer, $h_{\textrm{fuzz}}$, could be sorted in terms of the He flux, $\Phi _{\textrm{He}}$, even at different He fluxes, and it is empirically known as

2 $$\begin{aligned} h_{\textrm{fuzz}} \propto \sqrt{\Phi _{\textrm{He}}}. \end{aligned}$$

Thus, it is likely that the LFN growth can also be sorted by $\Phi _{\textrm{He}}$. However, if we rearrange Fig. 6 in terms of He fluence, taking into account the edge effect (Fig. 7d), four curves are not united but are separated into two groups: plate/$\sharp 290$ mesh and $\sharp 50$/$\sharp 100$ mesh. In Fig. 7d, the formed area started to increase from $\sim 1\times 10^{25}$ $\hbox {m}^{-2}$ on the plate and $\sharp 290$ mesh, while it started to increase from $2\hbox {-}3\times 10^{25}$ $\hbox {m}^{-2}$ on $\sharp 50$ and $\sharp 100$ meshes. The results suggest that there is another factor that fully explains the experimental results. In addition to He, the amount of W particles reaching the surface should be considered. The mean free path of W atoms from the sputtering wire was estimated to be 24 mm for a typical density of $2.3\times 10^{18}$ $\hbox {m}^{-3}$, considering the ionization rate coefficient of $5\times 10^{-14}$ $\hbox {m}^3/\hbox {s}$ at $\sim 5$ eV and the mean energy of 7.5 eV^46^. Although there is uncertainty about how much of the W atom is slowed by collisions before ionization, W neutrals are likely the dominant species, because the mean free path is estimated to be much longer than the distance from the sputtering wire to the sample. Thus, unlike He ions, the W flux will not be affected or concentrated by the electric field formed around the sample. In other words, the ratio of W/He flux will decrease at $\sharp 50/\sharp 100$ as the He flux increases. It is likely that the difference in the W/He flux ratio results in the shift of the onset He fluence to form LFNs, as shown in Fig. 7d. Finally, regarding the onset He fluence to form LFNs in Fig. 7d, it is interesting to note that the value is on the order of $10^{25}$ $\hbox {m}^{-2}$, although it can vary with changing the W/He flux ratio. The dependence of the W/He ratio is also clearly shown in Fig. 5a–c, where the LFN formed area decreases with increasing the section number. Because the wire is located on the left-hand side of the sample and the sputtered W density decreases with increasing the distance from the wire, the W/He ratio decreases from $\sharp 1$ to $\sharp 5$.

For fuzz, it is known as an incubation He fluence^24^, and the value is an order of magnitude less (1-$4\times 10 ^{24}$ $\hbox {m}^{-2}$)^30^ than that shown in Fig.7d. This is likely because the growth of tall nanofibers is necessary for the initial growth of LFNs, and fuzz formation is thought to be a prerequisite for nanofiber formation^31,33^.

### Co-deposition on NTB

#### Growth of NTBs

**Figure 8:**
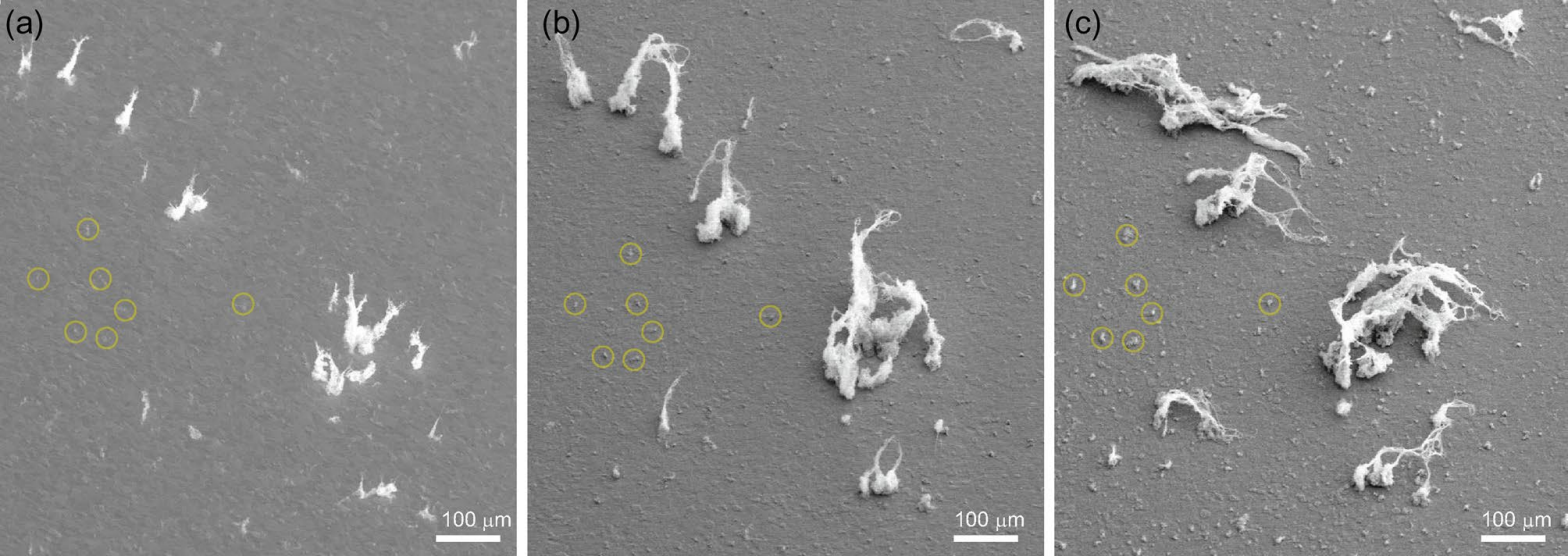
SEM images of (**a**) the NTB-formed W sample surface and (**b**,**c**) after He–W co-deposition for (**b**) 15 min and (**c**) 30 min.

Here, samples with NTBs prepared by exposure to He plasma with a mixture of a small amount of Ne are used to demonstrate and further investigate the edge effects. Figure 8a is an SEM image of the NTB-formed W sample surface. We have chosen this area to observe the variation of NTBs from the central region, excluding the left part which can be influenced by the LFNs grown from the edge. More than ten NTBs can be identified in the image, and the maximum height is $\sim 100$ $\upmu \hbox {m}$. Figure 8b,c shows the surface after He–W co-deposition was performed on the NTB-formed sample for 15 and 30 min, respectively. The first 15-min of He–W co-deposition resulted in significant growth of the NTBs. The height was almost doubled for most of the NTBs. Interestingly, NTBs in several locations were connected to form loop structures. It is thought that this was not a coincidence and that the Lorentz force between the two NTBs formed an attractive force to form the loops. After another 15 min He–W co-deposition, the NTB grew to lie on the W-substrate and spread on the surface, as shown in Fig. 8c. Fibers are entangled and form a thicker fiber.

This growth process was similar to the initial growth process of LFNs starting from the edges of the samples^33^, where fine structures grew in the height direction up to the height of $\sim$ 0.1 mm, and then, they started to expand in the surface direction. The NTBs were tilted toward the lower right direction in Fig. 8c, which is consistent with the previously observed growth direction of LFNs, and this is consistent with the direction of the plasma flow, i.e., a combination of parallel flow along with the magnetic field line and ${{\varvec{E}}} \times {{\varvec{B}}}$ drift.

During the 30 min of He–W co-deposition, the roughness significantly increased. For example, protrusions formed in the areas marked with yellow circles, although only white dots can be seen in Fig. 8a before the He–W co-deposition. In the later analysis with CLSM, no NTBs were identified at 0 min irradiation in the circled area, but NTBs were identified after 30 min of He–W co-deposition.

**Figure 9:**
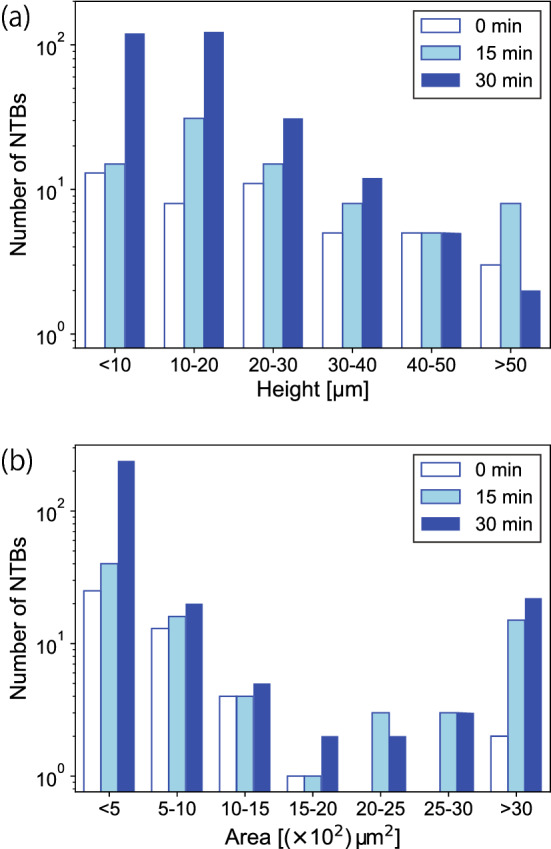
Evolution of (**a**) the height and (**b**) area distribution of NTBs during the exposure to He plasma with W co-deposition measured by CLSM.

To investigate this phenomenon quantitatively, the time evolution of the height and area of NTBs was measured using CLSM. It is difficult for CLSM to identify the tip of NTBs due to their fineness; here, we focus on the average height and area of NTBs, and the growth of the tip will be shown later. Also, only the central region ($4\times 4$ $\hbox {cm}^{2}$) of the sample was analyzed to focus on the growth of NTBs, as the growth of LFNs from the left edge was excluded. The evolution of the height and area distributions of NTBs are shown in Fig. 9(a,b), respectively. By the He–W co-deposition, the number of NTBs increased from 46 to 76 (15 min) and to 280 (30 min).

Before He–W co-deposition (0 min), the number of NTBs is small, and the height distribution is broad. After 15 min of He–W co-deposition, the distribution has a peak in the range of 10–20 $\upmu \hbox {m}$, and the number of NTBs increased in the whole height range shown in Fig. 9a. There were $\sim 10$ NTBs with a height greater than 50 $\upmu \hbox {m}$. After 30 min of He–W co-deposition, the number of NTBs smaller than 10 $\upmu \hbox {m}$ increased significantly and has a peak in this range. The distribution became sharper than that without and with 15 min of He–W co-deposition. The size of NTBs also increased with He–W co-deposition. The peak in size always had a maximum at 0–5 $\times 10^2$ $\upmu \hbox {m}^2$. The number of NTBs larger than $1.5\times 10^3$ $\upmu \hbox {m}^2$ increased significantly. Initially, there were only two NTBs larger than $2\times 10^3$ $\upmu \hbox {m}^2$, but these large NTBs increased to 21 at 15 min and 27 at 30 min of He–W co-deposition.

#### Growth rate of NTBs

**Figure 10:**
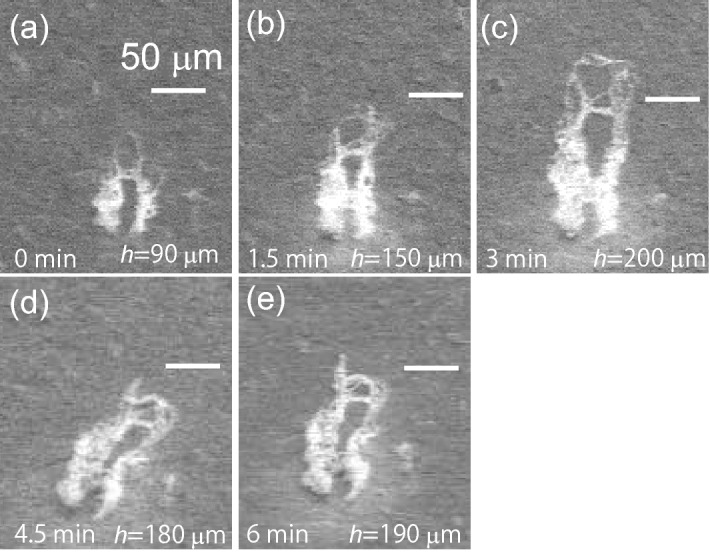
Evolution of an NTB by the co-deposition experiments (**a**) before co-deposition experiments, after **(b**) 1.5-min-irradiation, (**c**) 3.0-min-irradiation, (**d**) 4.5-min-irradiation, and (**e**) 6-min-irradiation.

To understand the growth of NTBs, including their tip, the growth rate was examined using SEM micrographs using the method described. As shown in Fig. 8, the LFNs grew significantly at 15-min intervals and grew as if lying on the substrate. Therefore, to focus on the tip growth in the height direction, plasma irradiation was set to 1.5-min intervals, and plasma irradiation and SEM observations were repeated. Figure 10 shows the growth of an NTB by the co-deposition experiments. The NTB clearly grew only with 1.5-min irradiation (Fig. 10a,b and b,c). The assessed heights in Fig. 10a–c were 0.9, 1.5, and $2.0\times 10^2$ $\upmu \hbox {m}$, respectively. During the three minutes of irradiation, the NTB grew to more than double. However, starting from 4.5 min (Fig. 10d,e), the structure tilted, and the estimated height decreased to $1.8\times 10^2$ $\upmu \hbox {m}$ at 4.5 min and $1.9\times 10^2$ $\upmu \hbox {m}$ at 6 min. Thus, when the growth reached a certain height, the growth direction could be changed, or the structure could be influenced by the plasma flow, as was discussed.

To discuss the growth characteristic statistically, the heights of NTBs in the $4\times 4$ $\hbox {mm}^2$ area of the central part of the sample, excluding edges, were measured, and the changes in the height were followed. We eliminated the data after the negative growth occurred due to the tilting of the structure, as shown in Fig. 10. We repeatedly performed 1.5 min irradiation and SEM observation, and the growth of NTBs was followed up to 6 min. However, the number of NTBs that continued to grow without negative growth decreased from 31 (0–1.5 min) to 16 (1.5–3 min), 11 (3–4.5 min), and 4 (4.5–6 min); the data up to 4.5 min were used.

**Figure 11:**
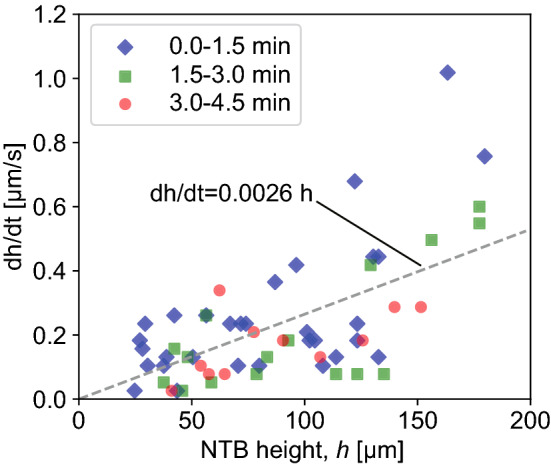
Growth rate of the NTB height as a function of the NTB height measured from SEM micrographs. The growth rate was derived from the variation in height before and after repeated 1.5 min of He irradiation under co-deposition conditions.

The growth rate of the height of NTBs, *dh*/*dt*, was derived from the change in height during 1.5-min of irradiation and plotted as a function of the height averaged before and after the 1.5-min of irradiation (Fig. 11). The three time intervals of 0–1.5 min, 1.5–3 min, and 3–4.5 min are plotted with blue, green, and red markers, respectively. Although there is scatter, *dh*/*dt* has an increasing trend with *h*. The growth rate of the NTB with $h<100~\upmu \hbox {m}$ was $0.16\pm 0.10$ $\upmu \hbox {m/s}$. In addition, when $h>100~\upmu \hbox {m}$, *dh*/*dt* was $0.33\pm 0.25$ $\upmu \hbox {m/s}$. Typically, the height of NTBs is several tens of $\mu \hbox {m}$ with an irradiation time of one hour^25,26^; the growth rate of NTBs without co-deposition is said to be on the order of 0.01 $\upmu \hbox {m/s}$. With He–W co-deposition, the growth rate increased by an order of magnitude. For LFN growth, the height was obtained empirically as^33^

3 $$\begin{aligned} h=a \exp (bt), \end{aligned}$$

where the coefficients for *a* and *b* were 0.13 $\upmu \hbox {m}$ and $0.0109\pm 0.006$ 1/s, respectively. From Eq. (3), we can obtain the following relation

4 $$\begin{aligned} \frac{dh}{dt}=ab \exp (bt)=bh. \end{aligned}$$

In Fig. 10, we fitted the measured *dh*/*dt* with a linear function of *h* to compare with the previous study. From the slope, it was assessed that $b=0.0026$ 1/s, which is a quarter of the value reported in Ref.^33^. The result indicated that the growth rate was four times less than the LFN growth on the edge of the sample, while it was an order of magnitude larger than that of NTBs.

Considering the fact that the averaged He flux was almost the same between the two experiments (slightly less than $10^{22}$ $\hbox {m}^{-2}\hbox {s}^{-1}$), one of the differences was in the W flux. In Ref.^33^, the wire bias was $\sim 50$ V negatively deeper than in the present study, so the sputtering rate should be $\sim 20\%$ higher than that in the present study. In addition, because the left edge of the sample is closer to the sputtering wire, the deposition rate of W at the left edge is greater than that at the left side of the sample.

## Conclusions

In this study, W plates and several W meshes with different mesh openings were exposed to He plasma under He–W co-deposition conditions. The growth of LFNs occurred throughout the W meshes that have much larger mesh openings than the sheath thickness. The feature is quite different from a W plate, where LFN growth occurs only from the edge close to the sputtering source. In other words, the edge effect appeared on these mesh samples. On the meshes, an increase in He flux by a factor of 3–4 was observed, and the concentration of the He flux was thought to result in the acceleration of the LFN growth. On all the samples, some He fluence was necessary before starting the growth of LFNs. The incubation He fluence, which was identified on fuzz growth and known to be $\hbox {1-4}\times 10^{24}$ $\hbox {m}^{-2}$, was $\hbox {1-3}\times 10^{25}$ $\hbox {m}^{-2}$ for LFNs. Because the growth of LFNs requires the formation of tall nanowires and the growth of fuzz, LFNs require incubation fluence that is an order of magnitude higher than fuzz. It was also observed that the ratio of W to He flux has an influence on the incubation fluence.

Although a mesh is not used in fusion devices, the edge effects mentioned above can occur in various cases. In addition to the edge of castellated divertor tiles, one typical example is NTBs, which can grow to $\sim 100$ $\upmu \hbox {m}$ by the exposure to He plasma irradiation with a small amount of Ne. In this study, NTB-formed W plates were used as samples to investigate whether NTBs can be the origin of the LFN during He–W co-deposition, focusing on the growth of NTBs using CLSM and SEM. In the He–W co-deposition of a W sample with NTBs, significant growth of NTBs occurred during 15-min irradiation. It was confirmed that NTBs can be the growth origin of the LFNs. To focus on the initial growth process, we repeatedly performed He–W co-deposition for 1.5-min, and the growth rate was deduced from the observations with SEM. The growth rate of NTBs with $h<100$ $\upmu \hbox {m}$ was found to be $0.16\pm 0.10$ $\upmu \hbox {m/s}$. The growth rate of NTBs was one order of magnitude greater than the growth rate of NTBs without He–W co-deposition. Although the growth rate is lower than the initial growth rate of LFNs observed previously^33^, the difference can be mainly explained by the difference in W deposition rate due to a longer distance from the sputtering source. We plan to investigate the co-deposition effects in a higher density regime to demonstrate the divertor region in fusion reactors. In this study, $n_e$ was on the order of $10^{18}$ $\hbox {m}^{-3}$; it is of interest to investigate how the change in the sheath thickness and the ionization process of the released W will affect the results in a higher density regime.

## Data Availability

The datasets analysed during the current study are available from the corresponding author on reasonable request.

## References

[1] Yoshida N (2003). Microscopic damage of metals exposed to the helium discharges in triam-1m tokamak and its impact on hydrogen recycling process. Nucl. Fusion.

[2] Nishijima D, Ye M, Ohno N, Takamura S (2004). Formation mechanism of bubbles and holes on tungsten surface with low-energy and high-flux helium plasma irradiation in nagdis-ii. J. Nucl. Mater..

[3] Takamura S, Ohno N, Nishijima D, Kajita S (2006). Formation of nanostructured tungsten with arborescent shape due to helium plasma irradiation. Plasma Fusion Res..

[4] Baldwin, M. & Doerner, R. Helium induced nanoscopic morphology on tungsten under fusion relevant plasma conditions. *Nucl. Fusion* **48**, 035001 (2008). http://stacks.iop.org/0029-5515/48/035001.

[5] Kajita S, Takamura S, Ohno N (2009). Prompt ignition of a unipolar arc on helium irradiated tungsten. Nucl. Fusion.

[6] Kajita S, Ito AM, Ibano K (2022). Growth of fiberform nanostructures on metal surfaces by helium plasma irradiation. J. Appl. Phys..

[7] Temmerman GD, Hirai T, Pitts RA (2018). The influence of plasma–surface interaction on the performance of tungsten at the ITER divertor vertical targets. Plasma Phys. Controlled Fusion.

[8] Kajita S, Yagi T, Kobayashi K, Tokitani M, Ohno N (2016). Measurement of heat diffusion across fuzzy tungsten layer. Results Phys..

[9] Kajita S, Ohno N, Hirahata Y, Hiramatsu M (2013). Field emission property of nanostructured tungsten formed by helium plasma irradiation. Fusion Eng. Design.

[10] Hwangbo D, Kajita S, Ohno N, Sinelnikov D (2017). Field emission from metal surfaces irradiated with helium plasmas. IEEE Trans. Plasma Sci..

[11] Sinelnikov D (2019). Field emission from nanostructured tendril bundles. IEEE Trans. Plasma Sci..

[12] Kajita S, Ohno N, Yoshida N, Yoshihara R, Takamura S (2012). Arcing on tungsten subjected to helium and transients: Ignition conditions and erosion rates. Plasma Phys. Controlled Fusion.

[13] Aussems DUB, Nishijima D, Brandt C, Doerner RP, Cardozo NJL (2014). Spectroscopic characterization and imaging of laser- and unipolar arc-induced plasmas. J. Appl. Phys..

[14] Kajita S, Hwangbo D, Ohno N (2019). Ignition and behavior of arc spots on helium irradiated tungsten under fusion relevant condition. IEEE Trans. Plasma Sci..

[15] Nishijima D, Baldwin M, Doerner R, Yu J (2011). Sputtering properties of tungsten ‘fuzzy’ surfaces. J. Nucl. Mater..

[16] Nishijima D (2011). Effects of steady-state plasma exposure on tungsten surface cracking due to elm-like pulsed plasma bombardment. Fusion Sci. Technol..

[17] Xu X (2018). A real-time wearable uv-radiation monitor based on a high-performance p-cuzns/n-tio2 photodetector. Adv. Mater..

[18] Deng X, Li Z, Cao F, Hong E, Fang X (2023). Woven fibrous photodetectors for scalable uv optical communication device. Adv. Funct. Mater..

[19] Kimura Y (2020). Improved hydrogen gas sensing performance of wo3 films with fibrous nanostructured surface. Appl. Surf. Sci..

[20] Kajita S (2013). Helium plasma implantation on metals: Nanostructure formation and visible-light photocatalytic response. J. Appl. Phys..

[21] Komori K (2015). Sulfur k-edge XANES for methylene blue in photocatalytic reaction over WO3 nanomaterials. Nucl. Instrum. Methods Phys. Res. Sect. B Beam Interact. Mater. Atoms.

[22] de Respinis M (2013). Efficient plasma route to nanostructure materials: Case study on the use of m-wo3 for solar water splitting. ACS Appl. Mater. Interfaces.

[23] Feng S (2022). Photoelectrochemical properties of plasma-induced nanostructured tungsten oxide. Appl. Surf. Sci..

[24] Baldwin M, Doerner R, Nishijima D, Tokunaga K, Ueda Y (2009). The effects of high fluence mixed-species (deuterium, helium, beryllium) plasma interactions with tungsten. J. Nucl. Mater..

[25] Hwangbo D (2018). Growth of nano-tendril bundles on tungsten with impurity-rich he plasmas. Nucl. Fusion.

[26] Hwangbo D, Kajita S, Tanaka H, Ohno N (2019). Growth process of nano-tendril bundles with sputtered tungsten. Nucl. Mater. Energy.

[27] Woller, K., Whyte, D. & Wright, G. Impact of helium ion energy modulation on tungsten surface morphology and nano-tendril growth. *Nucl. Fusion* **57**, 066005 (2017). http://stacks.iop.org/0029-5515/57/i=6/a=066005.

[28] Woller KB, Whyte DG, Wright GM (2021). Crystallographic analysis of nano-tendril bundle vs fuzz growth on tungsten exposed to helicon wave-coupled helium plasma. J. Appl. Phys..

[29] Kajita S, Kawaguchi S, Ohno N, Yoshida N (2018). Enhanced growth of large-scale nanostructures with metallic ion precipitation in helium plasmas. Sci. Rep..

[30] Petty, T., Baldwin, M., Hasan, M., Doerner, R. & Bradley, J. Tungsten ‘fuzz’ growth re-examined: The dependence on ion fluence in non-erosive and erosive helium plasma. *Nucl. Fusion* **55**, 093033 (2015). http://stacks.iop.org/0029-5515/55/i=9/a=093033.

[31] Kajita S, Okuyama T, Tanaka H, Ohno N (2021). Growth of mo large-scale fiberform nanostructures. Plasma Fusion Res..

[32] Kajita S (2019). Helium-plasma-induced straight nanofiber growth on hcp metals. Acta Mater..

[33] Kajita S, Kawaguchi S, Yoshida N, Ohno N, Tanaka H (2018). Morphologies of co-depositing W layer formed during he plasma irradiation. Nucl. Fusion.

[34] Shen W, Kajita S, Tanaka H, Shi Q, Ohno N (2022). Fuzz growth process under he-w co-deposition conditions. Plasma Fusion Res..

[35] Kajita S (2021). Accelerated/reduced growth of tungsten fuzz by deposition of metals. J. Nucl. Materials.

[36] McCarthy P, Hwangbo D, Bilton M, Kajita S, Bradley JW (2020). Enhanced fuzzy tungsten growth in the presence of tungsten deposition. Nucl. Fusion.

[37] Schneider CA, Rasband WS, Eliceiri KW (2012). Nih image to imagej: 25 years of image analysis. Nat. Methods.

[38] Huang T, Yang G, Tang G (1979). A fast two-dimensional median filtering algorithm. IEEE Trans. Acoust. Speech Signal Process..

[39] McReynolds, T. & Blythe, D. Chapter 12—image processing techniques. In McReynolds, T. & Blythe, D. (eds.) *Advanced Graphics Programming Using OpenGL*, The Morgan Kaufmann Series in Computer Graphics, 211–245 (Morgan Kaufmann, 2005). https://www.sciencedirect.com/science/article/pii/B9781558606593500147.

[40] Otsu N (1979). A threshold selection method from gray-level histograms. IEEE Trans. Syst. Man Cybern..

[41] Ohno N (2013). Influence of crystal orientation on damages of tungsten exposed to helium plasma. J. Nucl. Mater..

[42] Parish CM, Wang K, Doerner RP, Baldwin MJ (2017). Grain orientations and grain boundaries in tungsten nonotendril fuzz grown under divertor-like conditions. Scripta Mater..

[43] Takamura S (2020). Crystallinity and grain distributions of fiber-formed nanostructure on tungsten surface with helium plasma exposure. Materialia.

[44] Baldwin, M. J., Dejarnac, R., Komm, M. & Doerner, R. P. Fuzz growth in the gaps of castellated w in pisces-a: Experiment and modeling. *Plasma Phys. Controlled Fusion* **59**, 064006 (2017). http://stacks.iop.org/0741-3335/59/i=6/a=064006.

[45] Lieberman M, Lichtenberg A (2005). Principles of Plasma Discharges and Materials Processing.

[46] Eckstein, W. Calculated sputtering, reflection and range values. *IPP* 9/132 (2002).

